# Illusory motion and vection induced by a printed static image under flickering ambient light at rates up to 100 Hz

**DOI:** 10.1177/20416695231223444

**Published:** 2024-01-02

**Authors:** Tomoaki Kozaki, Takeharu Seno, Akiyoshi Kitaoka

**Affiliations:** Fukuoka Women’s University, Fukuoka, Japan; Kyushu University, Fukuoka, Japan; Department of Psychology, Ritsumeikan University, Kyoto, Japan

**Keywords:** visual illusion, illusory motion, flickering light, light-emitting diode

## Abstract

Visual motion signals can produce self-motion perception known as vection in observers. Vection can be generated by illusory motions in the form of global expantion in still images as well as by visual motion signals. The perception of vection can be enhanced by flickering images at a rate of 5 Hz. This study examined the illusory motion and vection induced by a printed static image under flickering ambient light at rates up to 100 Hz. The perception of illusory motion and vection were enhanced by flickering ambient lights at 50, 75, and 100 Hz. The enhancement effect was higher for the flicker rates expected to be detectable by humans. The findings of this study suggest that alternating bright and dark signals to the cone receptors and primary visual cortex trigger perceptions of illusory motions.

Visual motion signals can produce self-motion perception in observers. Such a perception, also known as vection, can be generated by visual motion signals, such as moving luminance-defined dots and gratings (Brandt et al., 1973; Seno et al., 2009). A static image on a computer screen presenting the illusion of global expansion can also generate vection (Seno et al., 2013). Furthermore, alternating this illusory image with a gray blank image at 5 Hz on a computer screen produces stronger vection than the nonflickering condition (Seno et al., 2013). This screen flicker effect is consistent with previous findings that eye movements and blinks enhance illusory motions (Beer et al., 2008; Faubert & Herbert, 1999; Murakami et al., 2006; Otero-Millan et al., 2012). However, the causal mechanisms of this effect remain unclear. To provide additional evidence for enhancement effect of flicker condition, herein, we explore if flickering ambient light (an alternating bright and dark environment) also enhances the illusory motion and vection generated by a printed static image.

It is assumed that humans can detect flickers at rates below 50 to 80 Hz (Watson, 1986). This limit is known as the critical flicker frequency (CFF). However, when normal subjects and patients with X-linked retinoschisis (XLRS) were presented with flickering light at 96 Hz in a study, the normal subjects generated a synchronized electroretinogram (ERG) but no flicker ERG was obtained for the patients with XLRS (Alexander et al., 2000). The functional loss of cone receptors in patients with XLRS can be due to the disruption of intercell communication (split of the layers; schisis) in the retina (Condon et al., 1986). The flicker ERG finding suggests that cone receptors can respond to flickering light at a rate around 100 Hz. The steady-state visual-evoked potential, which mainly results from the primary visual cortex (V1) (Di Russo et al., 2007; Muller et al., 1997), is also evoked by flickering light up to 90 Hz (Herrmann, 2001). Illusory motions are perhaps linked to activities in V1 (Conway et al., 2005). This finding indicates that both human cone receptors and V1 are sensitive to flickering light at frequencies around 100 Hz. Furthermore, observers can detect flickering light at much higher rate (e.g., 1 kHz) during saccades (Roberts & Wilkins, 2013).

In experiment 1, we preliminarily evaluated the perception of illusory motions in a printed static image under flickering ambient light at rates of up to 100 Hz. We aimed to investigate if flickering ambient light can modulate the perception of illusory motion in a printed static image—more specifically, if this modulation is restricted to flicker rates below the CFF.

## Experiment 1: Illusory Motion in a Printed Static Image Under Different Flickering Ambient Light Conditions

### Method

#### Participants

Participants (26 females aged between 21 and 23 years) were recruited from Fukuoka Women's University (Fukuoka City, Japan). All participants provided informed consent, had normal or corrected-normal vision, and were devoid of known medical conditions.

#### Light Conditions

Under all ambient light conditions, the light was emitted by a white light-emitting diode (LED) positioned on a ceiling. Flickering lights were generated from the LED using a function generator (AFG-21005; RS Components, PRO, UK) and a custom-built pulse-width modulation circuit. The flicker rate was set to 50, 75, and 100 Hz, and the duty rate (bright-to-dark proportion) was maintained at 50%. The horizontal light intensity was maintained at 100 lx by a direct-current power supply (PMX35-3A; Kikusui Inc., Japan) with an illuminance meter (CL-70F, Konica Minolta Inc., Japan).

#### Experimental Procedure and Design

**Figure 1.**
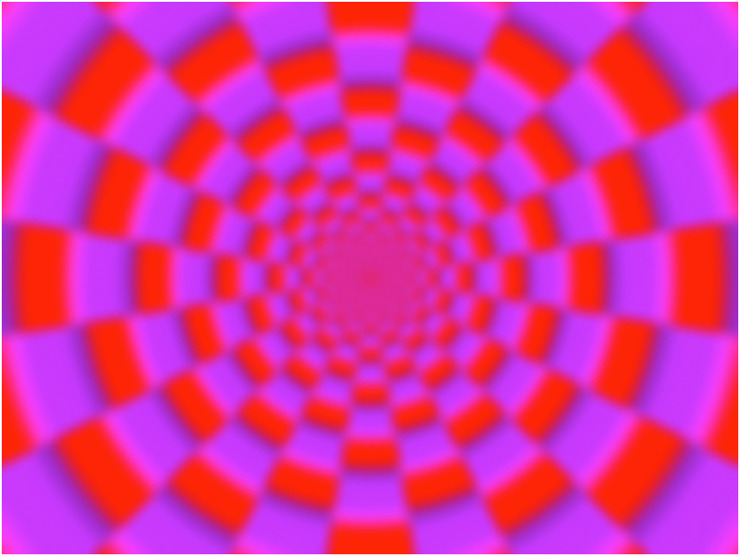
Printed static “Active Volcano” image. This image generates an illusory motion of global expansion in the participants.

The study elements were approved by the Research Ethics Committee of Fukuoka Women's University. First, the participants were placed on a chair in darkness in a windowless experimental chamber for 3 min. Subsequently, the strength of the illusory motion generated by a printed static image (41.0 cm  ×  56.5 cm; height  ×  width) was rated under nonflickering ambient light for 20 s (Figure 1). The printed static image was vertically placed on a desk facing the participants at 70 cm from the eyes. A printed static image with the same composition was used in an earlier study (Seno et al., 2013). After re-exposing the participants to darkness for 30 s, the strength of the illusory motion was rated under flickering ambient light for 20 s. To account for the effect of psychophysiological conditions (e.g., fatigue) on the oculomotor parameters (e.g., blink rate and saccade) of the participants (Lal & Craig, 2002; Schleicher et al., 2008), the strength of the illusory motion under each flickering condition was preliminarily measured under nonflickering ambient light as a baseline. Thereafter, the illusory motions under all flickering ambient light conditions were rated for 3 min of darkness, 20 s of nonflickering ambient light, 30 s of darkness, and 20 s of flickering ambient light. The strengths of the generated illusory motion under three flickering ambient light conditions (introduced in same order) were rerated by the participants after 10 min of rest in darkness. The strength of the generated illusory motion (not vection) in the image was rated on a 101-point scale ranging from 0 (no motion) to 100 (very strong motion).

#### Data Analysis

Prior to data analysis, the scores of the illusory motion strengths of each participant were averaged and statistically analyzed using the Statistics Package for Social Science (SPSS) version 29.0 (IBM SPSS, Chicago, IL, USA). A Shapiro–Wilk test revealed that the scores did not follow a normal distribution. The statistical significances of the differences in the scores of illusory motions between nonflickering and flickering light under each flickering ambient light condition were determined using a Friedman test with the Bonferroni–Holm correction. The amount of change in the scores between the nonflickering and flickering light were calculated for each flickering ambient light condition and analyzed using a Friedman test with the Bonferroni–Holm correction. A corrected *p* value of <.05 was considered statistically significant.

### Results

**Figure 2.**
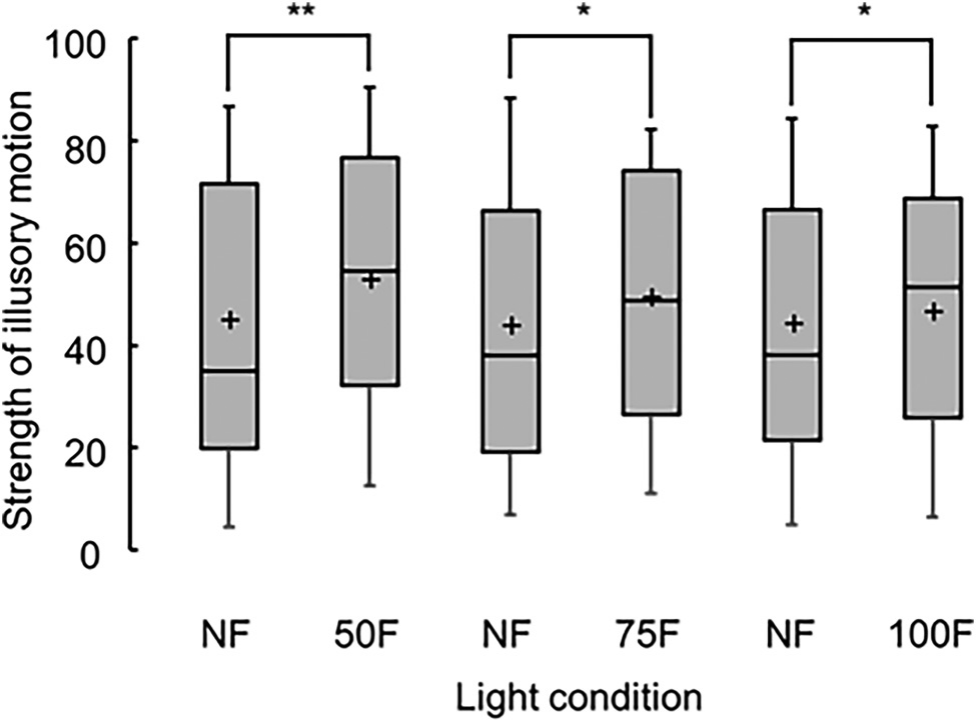
Boxplots of strengths of illusory motion in the Active Volcano image, showing the mean strengths ( + ), with significant differences of adjusted p being <.01 (**) and <.05 (*). Light conditions include nonflickering (NF) and flickering light at 50 (50F), 75 (75F), and 100 Hz (100F).

Figure 2 presents the scores of illusory motion strengths under each ambient light condition. The median scores under the nonflickering and flickering light were statistically different for all ambient light conditions: 35 and 55 under nonflickering and 50Hz flickering light, respectively (χ^2^(1)  =  25.0, adjusted *p* < .001); 38 and 49 under nonflickering and 75Hz flickering light, respectively (χ^2^(1)  =  6.0, adjusted *p*  =  .028); and 38 and 52 under nonflickering and 100Hz flickering light, respectively (χ^2^(1)  =  6.0, adjusted *p*  =  .014).

**Figure 3.**
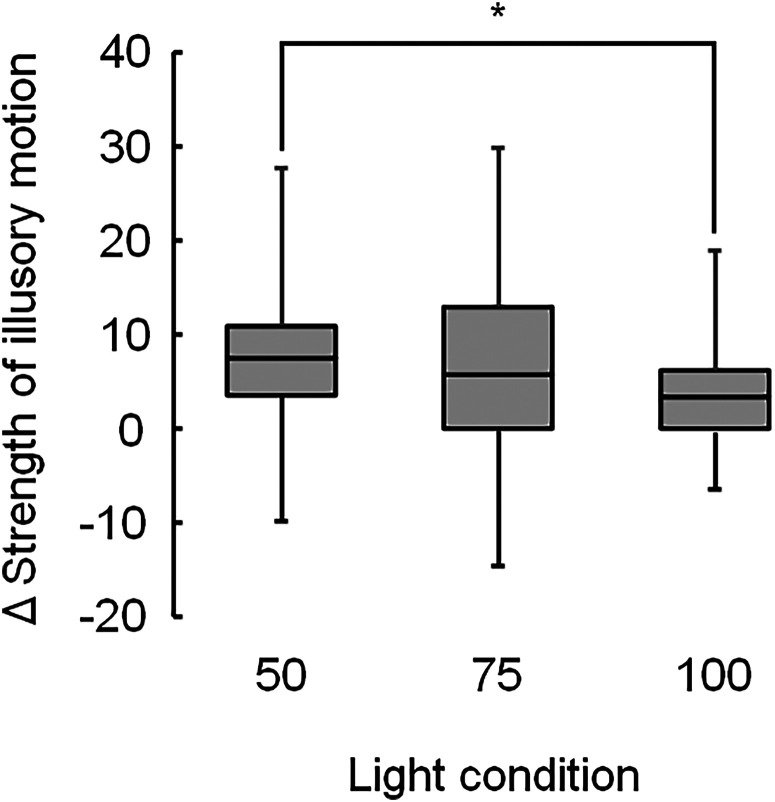
Changes in the strength of illusory motion between the flickering and nonflickering light for the Active Volcano image, showing the mean strengths ( + ), with significant differences of adjusted *p* being <.01 (**) and <.05 (*). Light conditions include flickering light at 50 (50), 75 (75), and 100 Hz (100).

The amount of change in the scores between the nonflickering and flickering light are depicted in Figure 3. The median score under 50Hz flickering light (7.5) was significantly higher than under 100Hz flickering light (3.0) (χ^2^(1)  =  9.0, adjusted *p*  =  .009).

### Discussion

For all flickering ambient light conditions, the median scores were substantially higher under flickering light than under nonflickering light. These findings suggest that flickering ambient light up to 100 Hz can enhance illusory motions in an image. Notably, the present findings indicate that flickering ambient light even above the CFF can influence illusory motions. Because human cone receptors and V1 are sensitive to flickering light at rates around 100 Hz (Alexander et al., 2000; Herrmann, 2001), alternating bright and dark signals may trigger and enhance the illusory motions.

Furthermore, the amount of change in the scores between the nonflickering and flickering light was higher for the 50Hz flickering ambient light condition than for the 100Hz flickering ambient light condition. The perception-enhancement effect of flickering ambient light might improve at human-detectable flicker rates. Human-detectable flickering light might also increase the blink rate because light stimuli (e.g., blight flashes) can induce the blink reflex (Rushworth, 1962).

In this experiment, the illusory motion was only evaluated in a printed static image. The image was designed based on a color-dependent Fraser–Wilcox illusion (Seno et al., 2013) called Active Volcano image. Furthermore, this experiment ignored the perception of self-motion, known as vection, generated by illusory motion in the image.

In experiment 2, we compared the perception-enhancement effects of flickering ambient light on Active Volcano and a matched control image. The control image was similar to Active Volcano image but generated no illusory motion (Seno et al., 2013). We also evaluated the vection generated by each image.

## Experiment 2: Illusory Motion and Vection in Printed Static Images Under Flickering Ambient Light Conditions

**Figure 4.**
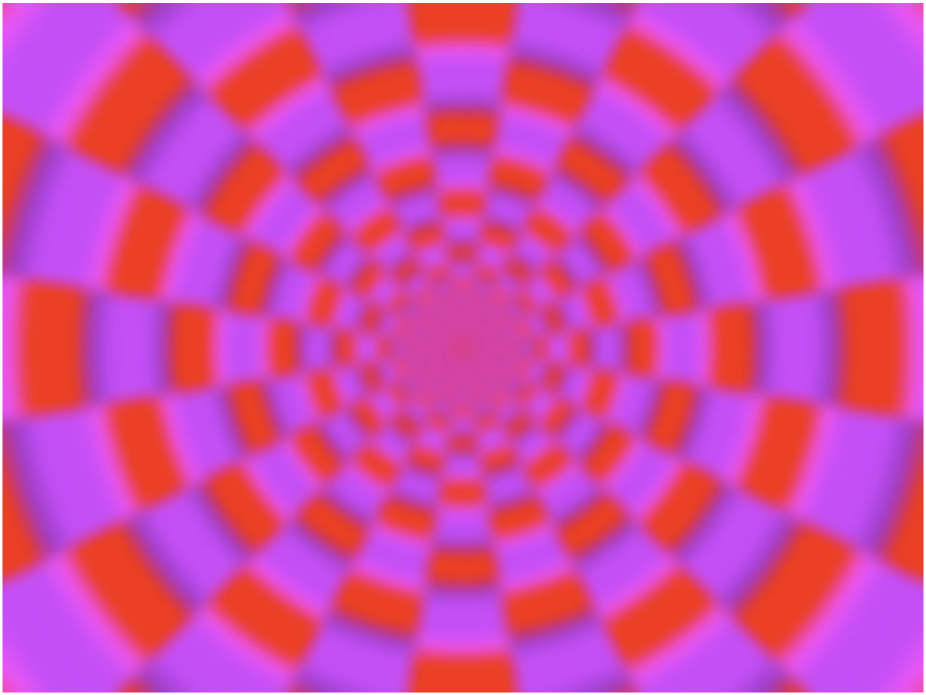
Printed static control image. This image is similar to the Active Volcano image but generates no illusory motion of global expansion.

During this experiment, we measured the strengths of the illusory motions in an Active Volcano image (Figure 1) and a matched control image (Figure 4). The control image generated no illusory motion in an earlier study (Seno et al., 2013). We also evaluated the strength of the vection induced by printed static images, where vection is defined as a visual illusion of self-motion in a stationary observer (Palmisano et al., 2015). As a baseline of vection, we measured the strength of the vection induced in a classical moving image.

### Method

#### Participants

The participants in this experiment included 13 females aged between 21 and 23 years. All the participants were recruited from Fukuoka Women's University (Fukuoka City, Japan), and provided informed consent, had normal or corrected-normal vision, and were devoid of known medical conditions.

#### Light Conditions

Flickering ambient light conditions were set to 50 and 100 Hz at a duty rate of 50%. All light conditions were implemented as in experiment 1.

#### Experimental Procedure and Design

**Figure 5.**
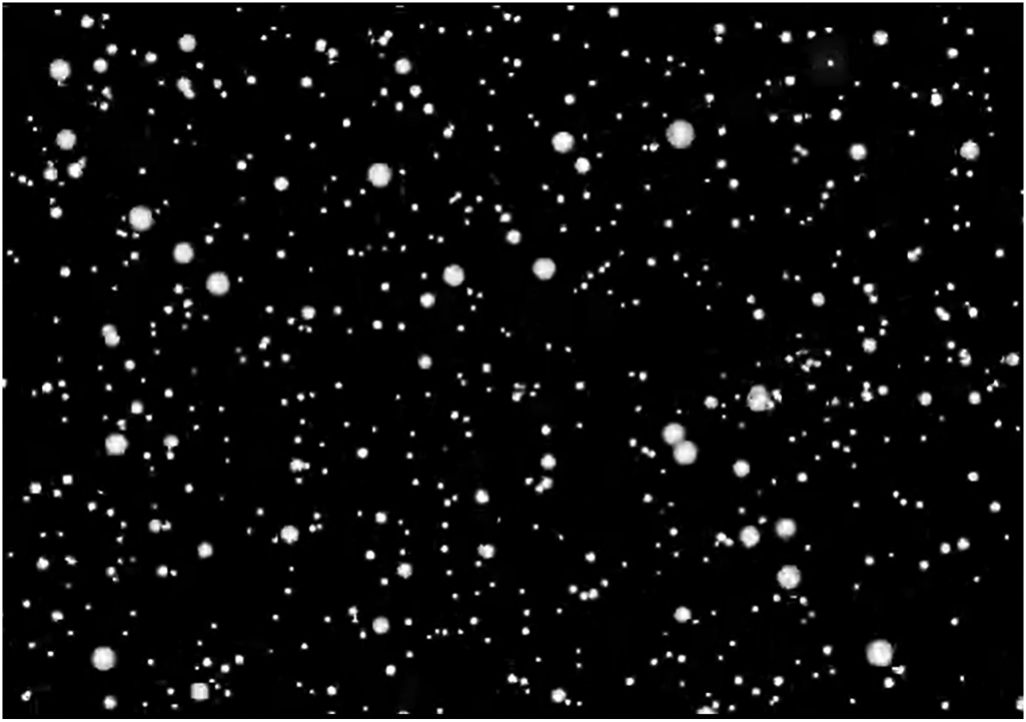
Moving image of global expansion dots. The moving image can be viewed at https://www.youtube.com/watch?v=vsQSnAgfCwY.

Prior to the experiment, the participants were instructed on the perception of vection and watched a moving image of global expansion dots (Figure 5). The moving image was a classical stimulus that can generate vection in an observer (Gibson, 1966). After instructions, the participants were placed on a chair in darkness in a windowless experimental chamber for 3 min. Subsequently, the participants rated the strength of the generated vection for 20 s (Figure 4). The moving image was presented on a 27-inch monitor (GW2780, BenQ Corp.) and a custom-built desktop computer. The monitor was placed in front of the participants at 50 cm from the eyes. The strength of the illusory motion and vection generated for the Active Volcano and control images were rated under flickering ambient light conditions as described in experiment 1 (3 min of darkness, 20 s of nonflickering light, 30 s of darkness, and 20 s of flickering light). The generated illusory motions in the images were rated on 101-point scales of motion ranging from 0 (no motion) to 100 (very strong motion) and vection ranging from 0 (no vection) to 100 (very strong vection).

#### Data Analysis

The data of this experiment were analyzed as described in experiment 1. All statistical analyses were conducted using SPSS version 29.0 (IBM SPSS, Chicago, IL, USA). A corrected *p* value of <.05 was considered statistically significant.

### Results

**Figure 6.**
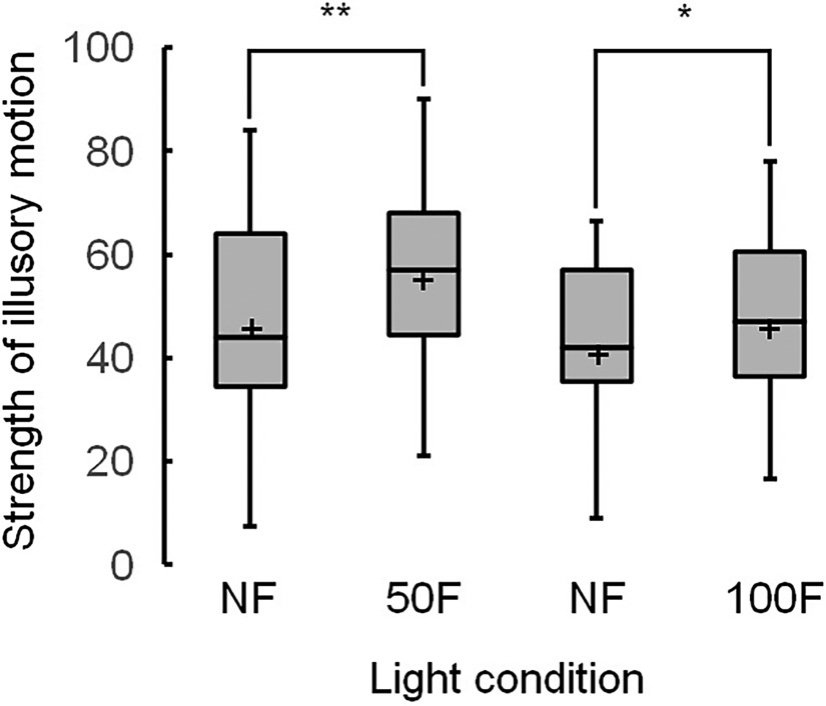
Boxplots of the strengths of illusory motion in the Active Volcano image, showing the mean strengths ( + ), with significant differences of *p* being <.01 (**) and <.05 (*). Light conditions include nonflickering (NF) and flickering light at 50 (50F) and 100 Hz (100F).

**Figure 7.**
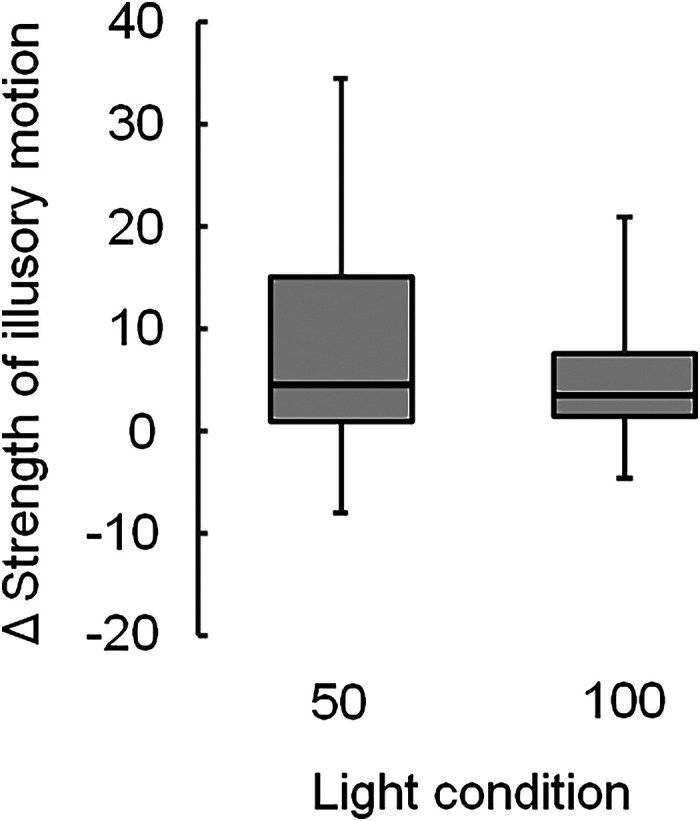
Changes in the strengths of illusory motion between the flickering and nonflickering light for the Active Volcano image, showing the mean strengths ( + ), with significant differences of adjusted *p* being <.01 (**) and <.05 (*). Light conditions include flickering light at 50 (50) and 100 Hz (100).

The strengths of illusory motion in the Active Volcano image under each ambient light condition are presented as boxplots in Figure 6. The median scores were significantly higher under flickering light than under nonflickering light: 57 versus 44 at 50 Hz ((χ^2^(1) = 8.3, adjusted *p*  =  .008) and 47 versus 42 at 100 Hz ((χ^2^(1)  =  6.2, adjusted *p*  =  .013), respectively. The amount of change in the scores between the flickering and nonflickering light are presented in Figure 7. The median scores were not significantly different between the 50 and 100Hz flickering light (4.5 and 3.5, respectively).

**Figure 8.**
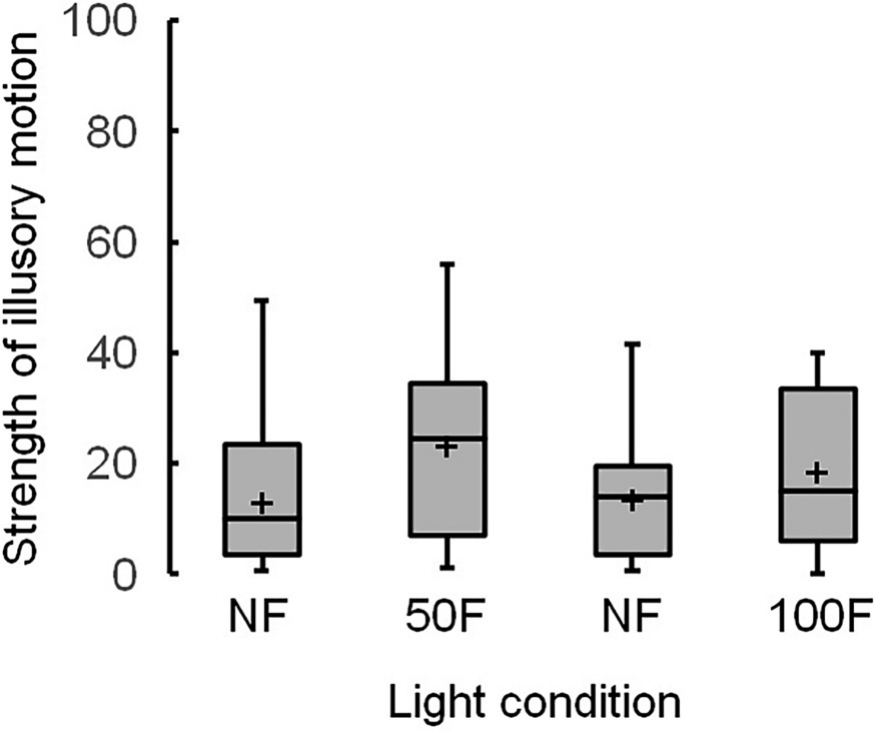
Boxplots of strengths of illusory motion in the control image, showing the mean strengths ( + ), with significant differences of *p* being <.01 (**) and <.05 (*). Light conditions include nonflickering (NF) and flickering light at 50 (50F) and 100 Hz (100F).

Figure 8 presents boxplots of the strengths of illusory motion in the control image. Again, no significant differences between the median scores of nonflickering and flickering light were observed at 50 and 100 Hz.

**Figure 9.**
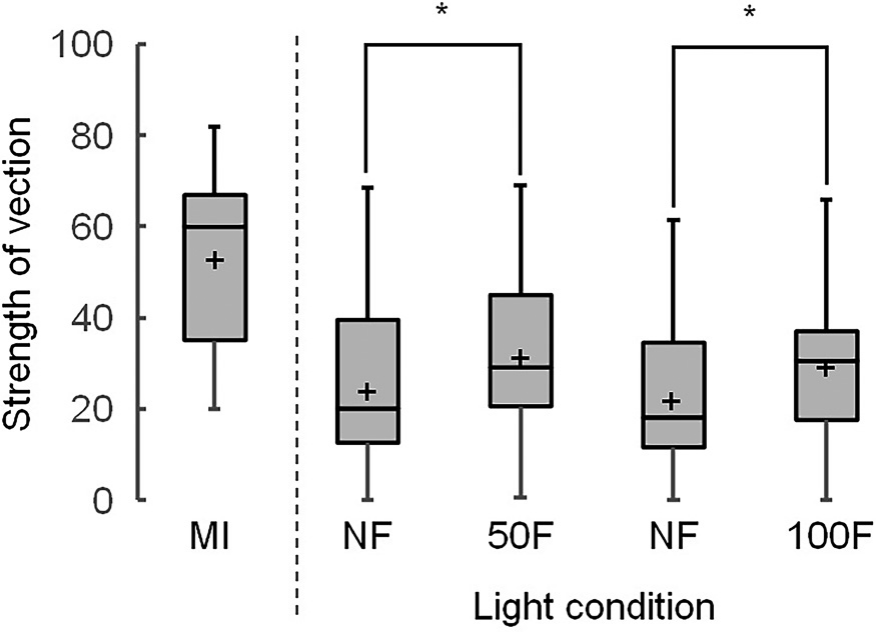
Boxplots of vection strengths in the moving image (MI) and Active Volcano image, showing the mean strengths ( + ), with significant differences of *p* being <.01 (**) and <.05 (*). Light conditions include nonflickering (NF) and flickering light at 50 (50F) and 100 Hz (100F).

**Figure 10.**
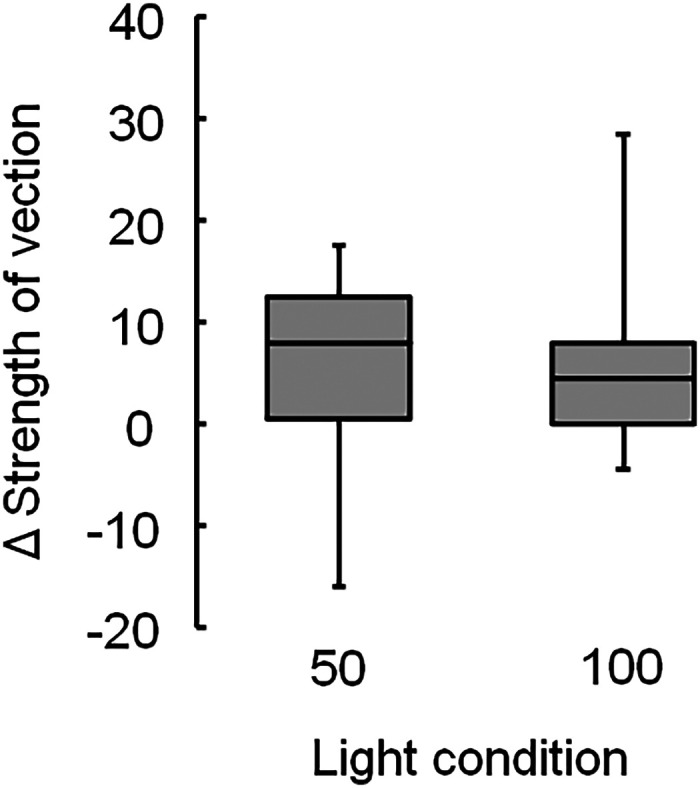
Changes in the vection strengths between the flickering and nonflickering light for the Active Volcano image, showing the mean strengths ( + ), with significant differences of adjusted *p* being <.01 (**) and <.05 (*). Light conditions include flickering light at 50 (50) and 100 Hz (100).

Figure 9 presents boxplots of the strengths of vection generated by the Active Volcano image under each ambient light condition. The median scores were significantly higher under flickering light than under nonflickering light: 29 versus 20 at 50 Hz ((χ^2^(1)  =  6.2, adjusted *p*  =  .026) and 31 versus 18 at 100 Hz ((χ^2^(1)  =  4.5, adjusted *p*  =  .035), respectively. The amount of change in the scores between the flickering and nonflickering light are presented in Figure 10. The median scores were not significantly different between the 50 and 100Hz flickering light (8.0 and 4.5, respectively).

**Figure 11.**
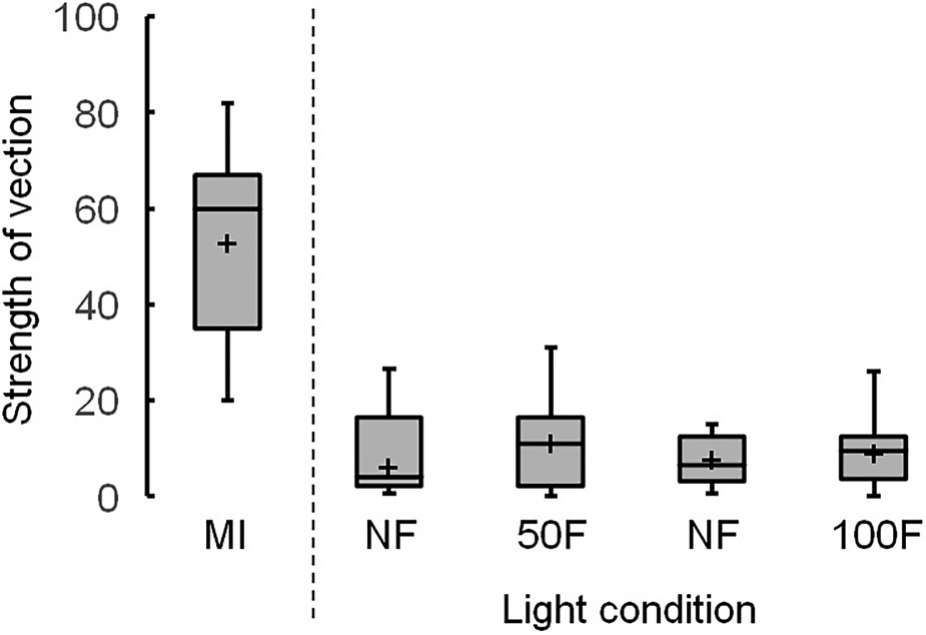
Boxplots of vection strengths in the moving image (MI) and control image, showing the mean strengths ( + ), with significant differences of *p* being <.01 (**) and <.05 (*). Light conditions include nonflickering (NF) and flickering light at 50 (50F) and 100 Hz (100F).

Figure 11 presents boxplots of the strengths of vection induced by the control image. No significant differences between nonflickering and flickering light were observed at 50 and 100 Hz. The median score of vection generated by the moving luminance-defined dots was 60.

### Discussion

The median scores of illusory motions at 50 and 100 Hz were higher in the Active Volcano image (44 and 42, respectively; Figure 6) than in the control image (14 and 10, respectively; Figure 8). Faubert and Herbert (1999) considered that motion perception in the Fraser–Wilcox illusion in the Active Volcano image depends on several conditions, including the luminance gradient that causes a difference in visual latency between the light and dark parts of the image. Brighter information can be processed faster than darker information in the visual system (Roufs, 1963; von Grunau et al., 1995; Wilson & Anstis, 1969). Kitaoka, who designed the Active Volcano image, asserted that the same configuration can generate different types of illusions, such as reverse-phi movement and position illusion (Kitaoka, 2006). He hypothesized that such configurations are elemental spatiotemporal configurations; one such example is a dark thin region flanked by a bright thick region and a middle-luminance thick region. The different processing speeds of visual information among luminance gradients can be regarded as a change in the location, generating illusory motion in the images.

In this experiment, flickering ambient light exerted no substantially enhancement on the illusory motion of the control image but enhanced the illusory motion of the Active Volcano image. The enhancement effects of flickering ambient light may depend on elemental spatiotemporal configurations of the image. As discussed for experiment 1, flickering ambient light with rates below the CFF might induce stronger illusory motions in the image than that with rates above the CFF. However, in this experiment, the amount of change in the scores between the flickering and nonflickering light showed no substantial difference between 50 and 100Hz flickering light. As the CFF depends on an observer's psychophysiological condition (e.g., fatigue) (Murata et al., 1996; Simonson & Enzer, 1941), some participants might have not detected 50Hz flickering light.

Under nonflickering light, the median scores of vection strength at 50 and 100 Hz were higher for the Active Volcano image (20 and 18, respectively; Figure 9) than for the control image (6.5 and 4, respectively; Figure 11). The Active Volcano image was designed to create a coherent illusory motion of global expansion. Because the participants were instructed that vection is a visual illusion of self-motion in a stationary observer (Palmisano et al., 2015) and watched a classical moving image (Gibson, 1966) before the experiment, they might have sensed vection in the Active Volcano image. These findings suggest that the illusory motion induced by the printed static Active Volcano image can generate vection similar to that of an image on a computer screen (Seno et al., 2013). However, the median scores of the Active Volcano image were lower than those of the classical moving image (60). The printed static image under nonflickering light did not induce stronger vection than the moving image.

Herein, the vection generated by the Active Volcano image was also enhanced by flickering lights with rates up to 100 Hz. The median scores of vection strength under 50Hz flickering light were not substantially different from those under 100Hz flickering light. As previously mentioned, some participants possibly did not detect 50Hz flickering light. The perception of vection might be substantially enhanced under human-detectable flickering ambient light conditions, as reported in an earlier study (Seno et al., 2013).

The alternative presentation of the images in the earlier study produced longer durations of illusory motions in the Active Volcano image (Seno et al., 2013). Therefore, vection under flickering ambient light can be enhanced not only by strengthening the illusory motion but also by extending the duration of light exposure. Furthermore, a previous study indicated that humans can perceive flicker artifacts on a display lit with an LED projector for rates up to 500 Hz if the display includes spatial edges (Davis et al., 2015). In some illusory images, such as those involving the Fraser–Wilcox illusion (Fraser & Wilcox, 1979), spatial edges comprise converging white and black lines. Flickering ambient light with rates above 100 Hz can enhance illusory motion and vection.

## Conclusion

This study suggests that ambient flickering light with rates up to 100 Hz can enhance illusory motion and vection in a printed static image. The enhancement effect of flickering ambient light appears to be improved when the observer can detect the flickering. In the experiments performed herein, the strengths of illusory motion and vection were first rated under nonflickering light and then under flickering light in each scenario. This procedure possibly induced a priming effect. Furthermore, flickering ambient light with rates above 100 Hz can amplify illusory motion and vection. Therefore, future studies should study illusory motions stimulated by other images under diverse flickering ambient light and experimental conditions.
